# Place of death and phenomenon of going home to die in Chinese adults: A prospective cohort study

**DOI:** 10.1016/j.lanwpc.2021.100301

**Published:** 2021-11-09

**Authors:** Li Weng, Yizhen Hu, Zhijia Sun, Canqing Yu, Yu Guo, Pei Pei, Ling Yang, Yiping Chen, Huaidong Du, Yuanjie Pang, Yan Lu, Junshi Chen, Zhengming Chen, Bin Du, Jun Lv, Liming Li

**Affiliations:** a Medical Intensive Care Unit, State Key Laboratory of Complex Severe and Rare Diseases, Peking Union Medical College Hospital, Peking Union Medical College, Chinese Academy of Medical Sciences, Beijing, 100730, China; b Department of Epidemiology & Biostatistics, School of Public Health, Peking University, Beijing 100191, China; c Peking University Center for Public Health and Epidemic Preparedness & Response, Beijing, China; d Fuwai Hospital, National Center for Cardiovascular Diseases, Chinese Academy of Medical Sciences and Peking Union Medical College, Beijing, China; e Chinese Academy of Medical Sciences, Beijing, China; f Medical Research Council Population Health Research Unit at the University of Oxford, Oxford, United Kingdom; g Clinical Trial Service Unit & Epidemiological Studies Unit (CTSU), Nuffield Department of Population Health, University of Oxford, United Kingdom; h Suzhou Center for Disease Control and Prevention, Jiangsu, China; i China National Center for Food Safety Risk Assessment, Beijing, China; j Key Laboratory of Molecular Cardiovascular Sciences (Peking University), Ministry of Education, Beijing, China

**Keywords:** place of death, going home to die, health insurance schemes, end-of-life, healthcare transition

## Abstract

**Background:**

China is embracing an ageing population without sustainable end-of-life care services. However, changes in place of death and trends of going home to die (GHTD) from the hospital remains unknown.

**Methods:**

A total of 42,956 participants from the China Kadoorie Biobank, a large Chinese cohort, who died between 2009 and 2017 was included into analysis. GHTD was defined as death at home within 7 days after discharge from the hospital. A modified Poisson regression was used to investigate temporal trends of the place of death and GHTD, and estimate prevalence ratios (PRs) and 95% confidence intervals (CIs) for the association of GHTD with health insurance (HI) schemes.

**Findings:**

From 2009 to 2017, home remained the most common place of death (71·5%), followed by the hospital (21·6%). The proportion of GHTD for Urban and Rural Residents’ Basic Medical Insurance (URRBMI) beneficiaries was around six times higher than that for Urban Employee Basic Medical Insurance (UEBMI) beneficiaries (66·0% vs 11·6%). Besides, a substantial increase in the proportion of GHTD throughout the study period was observed regardless of HI schemes (4·4% annually for URRBMI, and 5·4% for UEBMI). Compared with UEBMI beneficiaries, URRBMI beneficiaries were more likely to experience GHTD, with an adjusted PR (95% CI) of 1·19 (95% CI: 1·12, 1·27) (*P*<0·001).

**Interpretation:**

In China, most of deaths occurred at home, with a large proportion of decedents GHTD from the hospital, especially for URRBMI beneficiaries. Substantial variation in the phenomenon of GHTD across HI schemes indicates inequalities in end-of-life care utilization.

**Funding:**

The National Natural Science Foundation of China, the Kadoorie Charitable Foundation, the National Key R&D Program of China, the Chinese Ministry of Science and Technology.

**Research in context**

**Evidence before this study**

We searched PubMed for articles published before April 31, 2021, using terms related to the place of death (“place of death” OR “site of death” OR “hospital death” OR “home death”), and the phenomenon of going home to die from the hospital (“going home to die” OR “hospital to home”). We also searched for articles that had investigated the inequalities in end-of-life care between different health insurance schemes in China, using a combination of terms: (“health insurance”) AND (“inequality” OR “inequity” OR “disparities”) AND (“end-of-life”). No restrictions on study type or language were implemented. We manually searched reference lists and retrieved articles as well. Previous studies found that home was the most common place of death for the Chinese population. However, most studies have limitations such as selected geographic locations, older populations, or specific illnesses. Also, limited evidence is available about the extent to which going home to die from the hospital occurs in the wider Chinese population. Inequalities in healthcare utilization between different health insurance schemes have been extensively documented; however, whether such inequalities exist in end-of-life care utilization is unclear. Furthermore, whether the phenomenon of going home to die from the hospital impacts the in-hospital case fatality rate is worthy of further investigation.

**Added value of this study**

In this large population-based Chinese cohort, we found that the majority of deaths in China occurred at home, with a large proportion of decedents going home to die from the hospital between 2009 and 2017, especially for Urban and Rural Residents’ Basic Medical Insurance beneficiaries. A marked increase in the proportion of going home to die regardless of health insurance schemes was observed. Substantial variation in the phenomenon of going home to die across health insurance schemes indicates inequalities in end-of-life care utilization. In addition, neglecting the phenomenon of going home to die could lead to substantial underestimation of the in-hospital case fatality rate.

**Implications of all available evidence**

China is embracing an ageing population without sustainable end-of-life care services. Considering that home was the most common place of death and a larger proportion of decedents going home to die from the hospital, measures like building a high-quality primary healthcare delivery system, delivering community- or home-based end-of-life care services, and promoting the equity of end-of-life care might help meet the needs of rising ageing populations.

Alt-text: Unlabelled box

## Introduction

Place of death is an important quality marker for end-of-life (EOL) care at the population level. Studies in high-income countries suggested that home was the most preferred place of death.^1^ Similar findings were reported for terminal stage cancer patients in China.^2^ However, due to the cultural taboo of death, little evidence was available for the general attitude towards death and EOL care in the Chinese population.^3^ Although previous studies found that home was the most common place of death for the Chinese population, most studies are limited to selected geographic locations,^4^, ^5^, ^6^, ^7^ older populations,^4^,^8^ or specific illnesses.^4^,^6^

Current EOL care in China has limitations such as hospice care is provided mainly in inpatient settings^9^ and late-life hospitalization is increasing.^10^ Previous studies reported that ≥70% of deaths in China occurred at home^11^,^12^ with ≥90% of death certificates had medical visit history ^12^, which might due to a high proportion of decedents were transferring home to die. Similar findings were found in a tertiary medical centre.^7^ To our knowledge, little evidence is available about the extent to which going home to die (GHTD) occurs in the wider Chinese population. Furthermore, whether GHTD impacts the in-hospital case fatality rate (CFR), which is commonly used as a quality indicator of healthcare for hospitalized patients, is therefore well worth further investigation.^13^ As suggested, it would be better to capture the true level of CFR when deaths occurring within a given interval after hospital discharge were considered.^14^

Over the past decade, China's health system reform has made healthcare services more accessible and affordable for the whole population, with universal health insurance (HI) coverage by different HI schemes.^15^ In brief, the Urban Employee Basic Medical Insurance (UEBMI) covers working and retired employees in urban areas. The Urban Resident Basic Medical Insurance (URBMI) and New Rural Cooperative Medical Scheme (NRCMS) cover urban and rural residents, respectively, except for employees whom UEBMI should cover. These three schemes differed in the target population, administration, source and level of funding, and benefits, with UEBMI offering the most comprehensive service coverage and financial protections.^16^,^17^ The number of hospital beds (per 1000 people) increased dramatically from 2·3 in 2003 to 5·7 in 2017, accompanied by the hospital admission rate increasing 4·9 times from 3·6% in 2003 to 17·6% in 2017^15^. For patients with stroke and ischemic heart disease, Levy and colleagues reported reduced inequalities in case fatality among different HI schemes with increased hospital admission rates.^18^ However, with the expansion of hospital beds, little is known about changes in EOL care, especially about the occurrence of GHTD. It is also important and urgent to know whether the inequalities in EOL care between different HI schemes exist in the general Chinese population for delivering effective and sustainable EOL care services,^1^,^19^ considering the accelerated population ageing in China.

In this study, we aimed to describe the temporal patterns of EOL care and investigate the association between HI schemes and the phenomenon of GHTD in a cohort of 0·5 million Chinese adults, with the hope to provide evidence for a better EOL care policy in China. In addition, we estimated the extent to which in-hospital CFR was potentially underestimated when taking GHTD into account.

## Methods

### Data Sources

Details of design, methods, and population for the China Kadoorie Biobank (CKB) have been described elsewhere.^20^ Be brief, participants were recruited from ten study areas (5 urban and 5 rural areas) in China, aiming at maximizing geographic and socioeconomic diversity. Overall, 512,725 participants aged between 30 and 79 years were recruited between June 2004 and July 2008. All participants completed an interviewer-administered laptop-based questionnaire and physical measurements at baseline after signing a written informed consent (**Supplementary Methods**). Long-term follow-up for mortality was tracked electronically, by using unique personal identification numbers and following in the national Disease Surveillance Points system.^21^ The hospitalization records before death were obtained through the local National HI system. All fatal and nonfatal events were coded using the 10th revision of the International Classification of Diseases by trained staff blinded to the baseline data.

The study was approved by the Ethical Review Committee of the Chinese Center for Disease Control and Prevention (Beijing, China) and the Oxford Tropical Research Ethics Committee, University of Oxford (UK).

### Ascertainment of outcomes

The primary outcomes were the place of death and the EOL experience of GHTD from the hospital, which mainly reflected the EOL care of decedents in the last few weeks of life. The place of death was determined by the claim in the death certificate and grouped into four categories: home, hospital, emergency room (ER) or on the way to the hospital, and all other places (e.g., other cities, roads, and workplace). GHTD was defined as death at home within 7 days after discharge from the hospital. We classified decedents who received inpatient care in the last 7 days of life into two groups by their final place of death: GHTD and died in hospital (DIH). Decedents who were discharged from the hospital but died in places other than home were not included due to the small number of cases.

### Explanatory variables

The HI scheme for each participant had been identified annually through the HI system. Complying with the previous study,^18^ we imputed the missing data of the HI scheme for the year of death based on the closest scheme available between 2012 and 2016 (**Supplementary Methods**). The HI schemes were classified as UEBMI, URBMI, NRCMS, or uninsured (**Supplementary Methods**). In general, URBMI and NRCMS offer similar benefits.^16^ We, therefore, combined URBMI and NRCMS schemes as Urban and Rural Residents’ Basic Medical Insurance (URRBMI) in the present analyses.

### Assessment of covariates

Covariates collected or derived by questionnaire at baseline included age at death, sex, study areas, education, marital status, income, occupation, and household size. Besides, information on the underlying cause of death and the year of death obtained from the death certificate was also included. The underlying causes of death were grouped into six broad categories: malignant neoplasms (C00-C97), ischemic heart diseases (I20-I25), cerebrovascular diseases (I60-I69), diseases of the respiratory system (J00-J99), external causes (V01-Y98), and all other causes.

### Statistical analysis

**Fig. 1:**
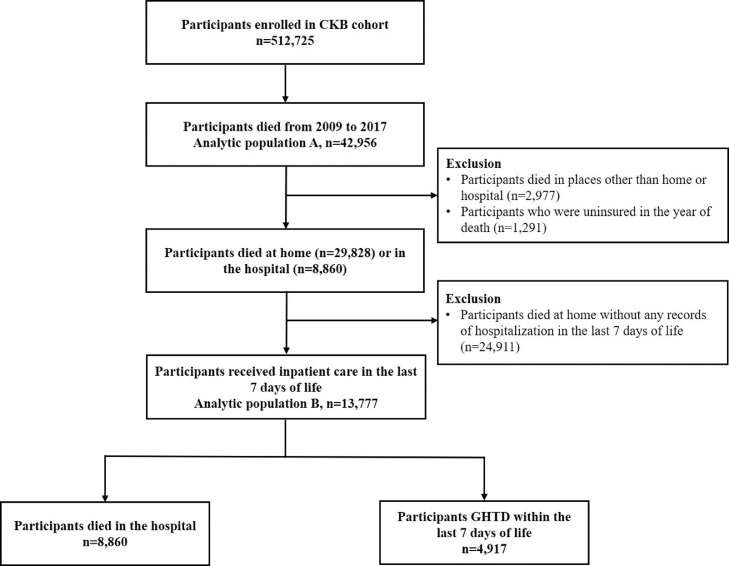
Flowchart illustrating the number of participants included in our study Abbreviations: CKB, China Kadoorie Biobank; GHTD: going home to die. Exclusion criteria were applied sequentially.

As the linkages to the HI system was not fully achieved until 2009, we, therefore, limited the study period to 2009 onwards. In the analysis of the temporal changes in place of death, we included all participants who died between 2009 and 2017 (analytic population A, n=42,956). In further analysis of GHTD, we sequentially excluded participants who died in places other than home or hospital (n=2,977), those who were uninsured in the year of death (n=1,291), and those who died at home without any records of hospitalization in the last 7 days of life (n=24,911). Finally, 13,777 decedents who received inpatient care in the last 7 days of life (analytic population B) were left, including the GHTD group of 4,917 decedents and the DIH group of 8,860 decedents (Fig. 1).

Because home death and GHTD phenomenon were relatively frequent, a modified Poisson regression with a robust error variance estimator was used to investigate temporal trends of the place of death and GHTD. The annual proportions and annual trends (percentage change per calendar year) of the place of death and GHTD were estimated overall and by the HI scheme (UEBMI or URRBMI) with adjustment for age at death, sex, and study area. Heterogeneity in trends in annual proportions of GHTD from the hospital between participants enrolled in different HI schemes was assessed using χ^2^ tests.

The modified Poisson regression was used to estimated prevalence ratios (PRs) and 95% confidence intervals (CIs) for the association of GHTD with HI schemes; such a method can mitigate an overestimate of the association caused by using odds ratio.^22^ The initial multivariate model (model 1) adjusted for age at death, sex, and study area. Model 2 further adjusted for socioeconomic factors including marital status, household income, education attainment, household size, and occupation. Model 3 adjusted for model 2 plus the underlying cause of death, and year of death. The analysis was also stratified by urban and rural residence because of substantial differences in access to healthcare and healthcare-seeking behaviour. The tests for interaction by residence were accessed using the likelihood-ratio tests, comparing model 3 with and without the interaction between urban or rural residence and the HI scheme.

According to previous studies,^23^ the calculation of inpatient quality indicators in the US,^24^ and the definition used in the Chinese Health Statistics Yearbook, in-hospital CFR was calculated by dividing the number of deaths that occurred in the hospital by the total number of hospital discharges plus the number of deaths happened in the hospital. The deaths that occurred in the ER were excluded. We reported the extent to which in-hospital CFR was potentially underestimated by dividing the number of decedents who met the definition of GHTD by the sum of decedents who met the definition of GHTD and those who died in the hospital.

Sensitivity analyses were conducted to investigate whether our findings of GHTD were robust to the alternative intervals (3 days, 14 days, or 30 days), which were used to define GHTD. We also performed association analysis between HI schemes and GHTD by stratifying the underlying cause of death considering the potential differences in healthcare-seeking behaviour between participants with different underlying causes of death. All statistical analyses were performed using Stata version 15·0. Statistical significance was determined at two-tailed *P* < 0·05.

### Role of the funding source

The funders had no role in the study design, data collection, data analysis and interpretation, writing of the report, or the decision to submit the article for publication.

## Results

### Place of death

Overall, 42,956 death was reported from the 512,725 CKB cohort participants between 2009 and 2017 (Fig. 1, analytic population A). The sociodemographic characteristics of the analytic population A were shown in **Table S1**. The mean age (SD) at death was 69·3 (10·0) years, and 43·7% were female. The most common place of death was home (71·5%), followed by the hospital (21·6%). The home deaths were more likely to occur in decedents from rural, had lower education level and annual household income, enrolled in the URRBMI scheme, or had larger household size. For deaths occurring at home, malignant neoplasms and cerebrovascular diseases were the most common causes of death (55·8%); for deaths occurring in hospital settings, malignant neoplasms accounted for 39·9% of all causes of death; for deaths occurring in ER or on the way to the hospital, ischemic heart diseases and external causes were the most common causes of deaths (49·4%).

**Fig. 2:**
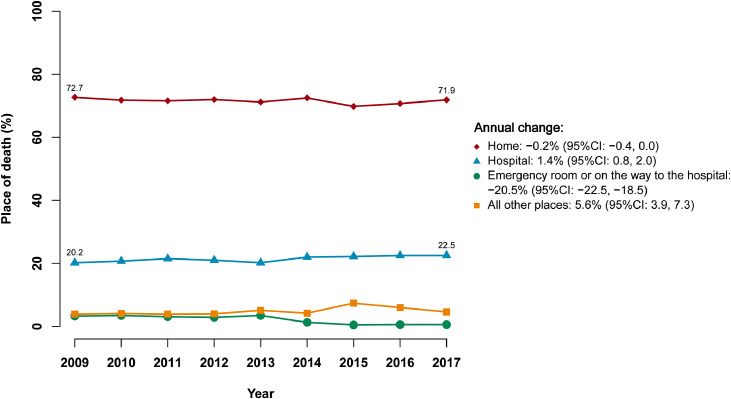
Changes in the proportion of place of death from 2009 to 2017 among 42956 decedents The Poisson models were adjusted for age at death, sex, and study area.

In analytic population A, the proportion of home death slightly changed from 72·7% in 2009 to 71·9% in 2017, with an annualized change of -0·2% (95% CI: -0·4%, 0·0%) (Fig. 2). There was a similar trend of home death among URRBMI beneficiaries, which changed by -0·2% (95% CI: -0·3%, 0·0%) annually from 89·4% in 2009 to 87·8% in 2017 (**Fig. S1**). On the contrary, among UEBMI beneficiaries, the proportion of home death increased by 1·4% (95% CI: 0·5%, 2·3%) annually from 34·2% in 2009 to 40·7% in 2017. Between 2009 and 2017, the proportion of hospital death among UEBMI beneficiaries increased by 1·4% (95% CI: 0·8%, 2·0%) annually, from 51·5% to 54·6%. However, no statistically significant change was observed in hospital death among URRBMI beneficiaries, which changed by 1·3% (95% CI: -0·3%, 2·8%) annually from 6·4% to 7·7%. Meanwhile, the proportion of deaths that occurred in the ER or on the way to hospital changed by -20·5% (95% CI: -22·5%, -18·5%) annually, and the proportion of death in all other places changed by 5·6% (95% CI: 3·9%, 7·3%) annually (Fig. 2).

Among all the 29,828 decedents who died at home, UEBMI beneficiaries received a higher proportion of inpatient services before death than their URRBMI counterparts (**Table S2**). The length of hospital stay of the last hospitalization before death was also longer for UEBMI beneficiaries than URRBMI.

### The phenomenon of GHTD

A total of 13,777 decedents received inpatient service within 7 days before death (Fig. 1, analytic population B), including 8,860 decedents who died in the hospital and 4,917 decedents GHTD based on our definition in the primary analysis. Decedents GHTD from the hospital were more likely to live in rural areas, have a lower level of education, have a lower level of annual household income, and have larger household size (**Table S3**). Also, GHTD was more common in decedents enrolled in the URRBMI than UEBMI (66·0% vs 11·6%) (Table 1). Although malignant neoplasms were the most common underlying causes of death for both groups of UEBMI and URRBMI, the proportion of malignant neoplasms was lower in the group of URRBMI than UEBMI (34·5% vs. 43·7%), and the proportion of cerebrovascular diseases and diseases of the respiratory system were higher in the group of URRBMI than in UEBMI (22·2% vs. 16·3%, 11·5% vs. 9·7%, respectively).

**Table 1:** Characteristics of 13777 CKB decedents who received inpatient care in the last 7 days of life according to the health insurance schemes.

	**Overall (n=13777)**		**Health insurance scheme^a^**			***P*-value^b^**
			**UEBMI (n=7671)**	**URRBMI (n=6106)**		
**Place of death, n (%)**						<0.001
Going home to die	4917 (35.7)		888 (11.6)	4029 (66.0)		
Died in hospital	8860 (64.3)		6783 (88.4)	2077 (34.0)		
**Age at death, year (SD)**	69.4 (9.8)		70.4 (9.7)	68.1 (9.9)		<0.001
**Sex, n (%)**						<0.001
Male	7865 (57.1)		4575 (59.6)	3290 (53.9)		
Female	5912 (42.9)		3096 (40.4)	2816 (46.1)		
**Place of residence, n (%)**						<0.001
Rural area	5070 (36.8)		436 (5.7)	4634 (75.9)		
Urban area	8707 (63.2)		7235 (94.3)	1472 (24.1)		
**Education level, n (%)**						<0.001
No formal school	2765 (20.1)		791 (10.3)	1974 (32.3)		
Primary and junior high school	8295 (60.2)		4463 (58.2)	3832 (62.8)		
Senior high school and above	2717 (19.7)		2417 (31.5)	300 (4.9)		
**Marital status, n (%)**						<0.001
Married	11497 (83.5)		6485 (84.5)	5012 (82.1)		
Others**^c^**	2280 (16.5)		1186 (15.5)	1094 (17.9)		
**Annual household income (RMB yuan), n (%)**						<0.001
<10000	3753 (27.2)		1008 (13.1)	2745 (45.0)		
10000-19999	4526 (32.9)		2903 (37.8)	1623 (26.6)		
20000-34999	3420 (24.8)		2362 (30.8)	1058 (17.3)		
≥35000	2078 (15.1)		1398 (18.2)	680 (11.1)		
**Occupation, n (%)**						<0.001
Managers or professionals	344 (2.5)		312 (4.1)	32 (0.5)		
Agricultural, manufacturing, services or sales workers	5071 (36.8)		1259 (16.4)	3812 (62.4)		
Other occupations, housework, retired, or unemployed	8362 (60.7)		6100 (79.5)	2262 (37.0)		
**Household size, n (%)**						<0.001
Live alone	981 (7.1)		563 (7.3)	418 (6.8)		
2 people	4762 (34.6)		3162 (41.2)	1600 (26.2)		
3-4 people	4550 (33.0)		2833 (36.9)	1717 (28.1)		
≥5 people	3484 (25.3)		1113 (14.5)	2371 (38.8)		
**Underlying cause of death, n (%)**						<0.001
Malignant neoplasms	5458 (39.6)		3353 (43.7)	2105 (34.5)		
Ischemic heart diseases	1876 (13.6)		1102 (14.4)	774 (12.7)		
Cerebrovascular diseases	2609 (18.9)		1251 (16.3)	1358 (22.2)		
Diseases of the respiratory system	1445 (10.5)		741 (9.7)	704 (11.5)		
External causes	470 (3.4)		167 (2.2)	303 (5.0)		
Other causes	1919 (13.9)		1057 (13.8)	862 (14.1)		

Abbreviations: CKB, China Kadoorie Biobank; UEBMI: Urban Employee Basic Medical Insurance; URRBMI: Urban and Rural Residents’ Basic Medical Insurance; SD, standard deviation.

Column percentages were provided.

^a^Information on health insurance scheme is for the year of death.

^b^Comparisons between groups were made using the ANOVA test for continuous variables and the Chi-Squared test for categorical variables.

^c^Including widowed, divorced or separated, or never married.

**Fig. 3:**
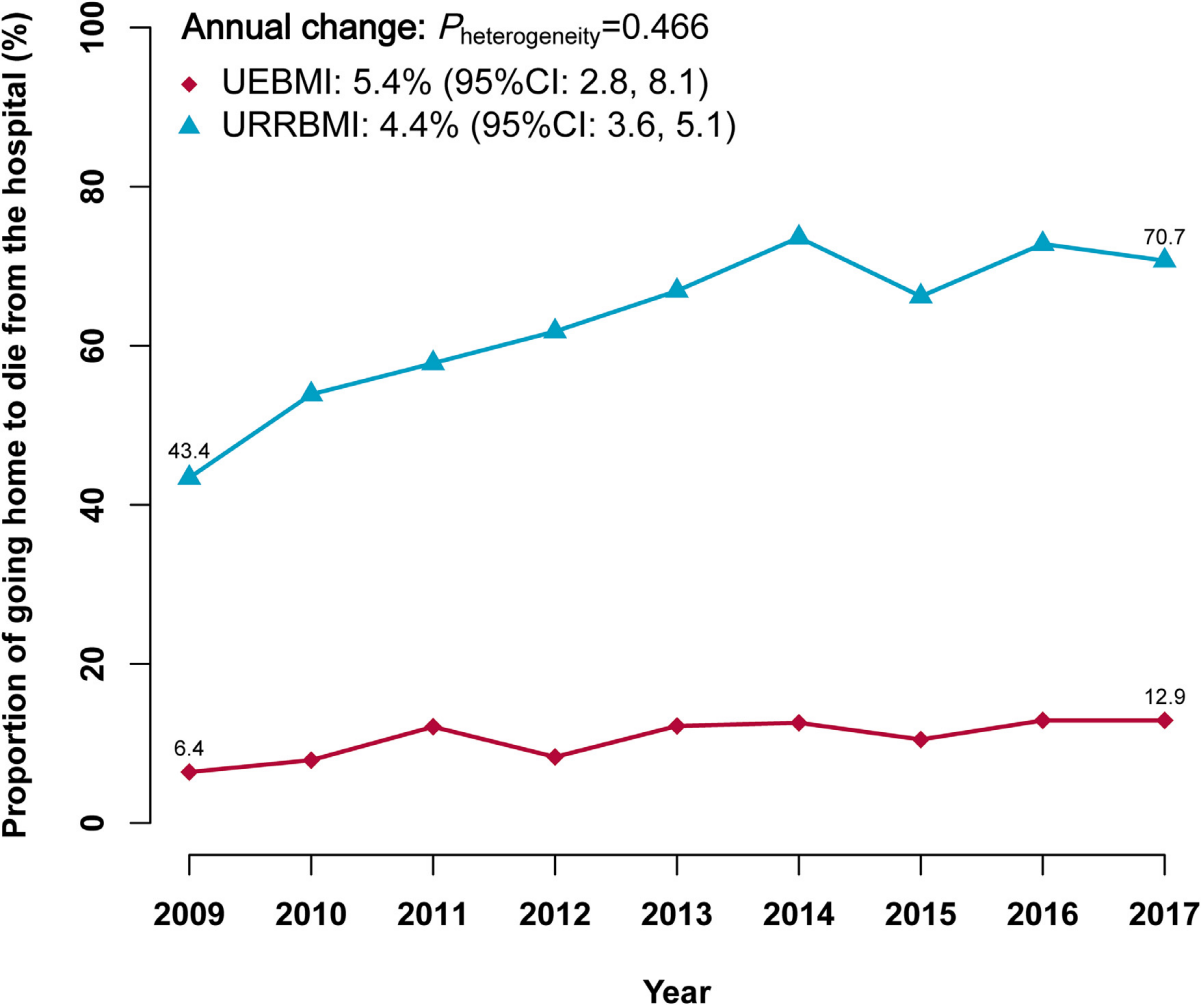
Changes in the proportion of going home to die from the hospital from 2009 to 2017 among 13777 decedents who received inpatient care within the last 7 days of life Abbreviations: UEBMI: Urban Employee Basic Medical Insurance; URRBMI: Urban and Rural Residents’ Basic Medical Insurance. Information on health insurance scheme is for the year of death. The Poisson models were adjusted for age at death, sex, and study area.

Throughout the study period, we noted a marked increase in age-, sex-, and area-adjusted proportion of GHTD in URRBMI beneficiaries, from 43·4% in 2009 to 70·7% in 2017 (4·4% annually, 95% CI: 3·6%, 5·1%) (Fig. 3). The corresponding proportion of GHTD in UEBMI beneficiaries also showed a substantially increasing trend, from 6·4% in 2009 to 12·9% in 2017 (5·4% annually, 95% CI 2·8%, 8·1%).

### Association between HI schemes and GHTD phenomenon

A statistically significant association was found between HI schemes and GHTD phenomenon (Table 2). Compared with UEBMI beneficiaries, URRBMI beneficiaries were more likely to experience GHTD, with an unadjusted PR of 5·70 (95% CI: 5·34, 6·08; *P*<0·001). The PR was substantially attenuated after adjustment for age, sex, and area (model 1), and was further attenuated following additional adjustment for socioeconomic factors (model 2) [1·28 (95% CI: 1·20,1·36; p<0·001), 1·17 (95% CI: 1·10, 1·24; *P*<0·001), respectively]. The PR was 1·19 (95% CI: 1·12, 1·27; *P*<0·001) after adjustment for variables in model 2, the underlying cause of death, and year of death (model 3). When stratified according to the residence of decedents, such association was similar between urban and rural areas (Table 2, *P*_interaction_=0·904).

**Table 2:** PRs (95% CIs) for the likelihood of going home to die from the hospital associated with health insurance schemes among 13777 decedents who received inpatient care in the last 7 days of life.

	Overall (n=13777)		Urban areas (n=8707)		Rural areas (n=5070)	
	UEBMI	URRBMI	UEBMI	URRBMI	UEBMI	URRBMI
Decedents/GHTD, n/n	7671/888	6106/4029	7235/617	1472/551	436/271	4634/3478
GHTD, %	11.6	66.0	8.5	37.4	62.2	75.1
Unadjusted model						
PRs (95% CIs)	1.00	5.70 (5.34, 6.08)	1.00	4.39 (3.97, 4.85)	1.00	1.21 (1.12, 1.30)
-value	<0.001		<0.001		<0.001	
Model 1						
PRs (95% CIs)	1.00	1.28 (1.20, 1.36)	1.00	1.33 (1.21, 1.47)	1.00	1.22 (1.14, 1.32)
*P*-value	<0.001		<0.001		<0.001	
Model 2						
PRs (95% CIs)	1.00	1.17 (1.10, 1.24)	1.00	1.15 (1.04, 1.28)	1.00	1.13 (1.05, 1.22)
*P*-value	<0.001		0.006		0.002	
Model 3^a^						
PRs (95% CIs)	1.00	1.19 (1.12, 1.27)	1.00	1.13 (1.02, 1.25)	1.00	1.22 (1.13, 1.31)
*P*-value	<0.001		0.024		<0.001	

Abbreviations: PR, prevalence ratio; CI, confidence interval; UEBMI: Urban Employee Basic Medical Insurance; URRBMI: Urban and Rural Residents’ Basic Medical Insurance; GHTD, going home to die.

Information on health insurance scheme is for the year of death. The modified Poisson models were used. Model 1 was adjusted for age at death (years), sex (male or female), and ten study areas; model 2 was further adjusted for marital status (married or others), household income (<10000, 10000-19999, 20000-34999, or ≥35000 RMB yuan), education attainment (no formal school, primary and junior high school, or senior high school and above), household size including self (1, 2, 3-4, or ≥5), occupation (managers or professionals, agricultural, manufacturing, services or sales workers, other occupations, housework, retired, or unemployed); model 3 was adjusted for model 2 plus the underlying cause of death, and year of death.

^a^The interaction p-value for statistical interaction between the place of residence (urban and rural areas) and the health Uncited References:insurance scheme was 0.904.

### Influence of GHTD on in-hospital CFR

Between 2009 and 2017, the number of decedents who died in the hospital was 8,860. If only deaths during hospital stay were used to calculate CFR, but ignoring deaths at home post-discharge within 3, 7, 14, and 30 days, the corresponding degree of underestimation of CFR was 29·7%, 35·7%, 41·5%, and 48·1%, respectively (**Table S4)**.

### Sensitivity analyses

In the sensitivity analyses using the alternative intervals (3 days, 14 days, or 30 days) to define GHTD, the temporal trends in the proportion of GHTD were consistent with the main analysis (**Fig. S2**). We consistently observed an increased likelihood of GHTD associated with URRBMI whichever intervals were used to define GHTD (**Table S5**). However, rural decedents were more sensitive to the intervals used for definition, with shorter intervals having higher effect estimates. The point estimates from the multivariable model were essentially unchanged when stratified by the underlying cause of death, although for some subgroups the values should be considered with caution since the wide CIs were found (**Table S6**).

## Discussion

In this large population-based Chinese cohort, we found that home deaths accounted for a significant and constant proportion of all deaths that occurred throughout the study period. The GHTD phenomenon was common among decedents who received inpatient services before death and associated with HI schemes. There was a marked increase in the proportion of GHTD regardless of HI schemes. URRBMI beneficiaries were more likely to experience GHTD compared to UEBMI counterparts. To our knowledge, our study is the first attempt to describe the phenomenon of GHTD in the general Chinese population.

Although the deep-rooted traditional Chinese cultural values and beliefs, i.e., Confucianism with characteristics of filial piety and family staying-together, played an important role in determining the place of death, other factors, such as individual demographic and socioeconomic characteristics, personal ethnic affiliation, underlying disease, the healthcare system, practical and operational issues,^3^ also played a role. The observation for most of the deaths occurred at home in our study was consistent with previous studies conducted in the Chinese population.^4^,^5^,^7^,^8^,^11^ However, against the background of rapid expansion of hospital beds per capita and increased hospital admission rates,^15^,^18^ such a constant proportion of home deaths was unexpected. Previous research in high-income countries indicated that the place of death was influenced by the availability and accessibility of hospital beds,^25^, ^26^, ^27^ with more deaths would occur in hospitals with more available hospital beds, even though it was discordant with the patients’ preference. Indeed, we found that more than one-third of hospitalized end-stage participants were discharged to home within 7 days before death, which means the occurrence of GHTD. GHTD was called “self-discharged” in a previous study ^28^ and might explain the incongruity between the constant proportion of home deaths and expanded hospital beds partially. However, self-discharged generally refers to a patient choose to leave the hospital against medical advice and most of the self-discharged patients survived after being discharged.^29^ The inexplicit definition of self-discharged may explain the inconsistent findings of a study using the data from the National Health Services Survey, in which Meng and colleagues found the proportion of inpatient self-discharge decreased from 36·8% in 2008 to 31·8% in 2011.^30^

We found that the occurrence of GHTD was increasing rapidly throughout the study period. The place of death was commonly confused with the place of care at EOL.^31^ The process of GHTD implied that decedents were more likely to be cared for in the hospital and discharged home in a terminal stage within a few weeks before death. Without adequate quality and quantity of healthcare resources or social support, GHTD might be a feasible pathway to maintain both cultural integrity and qualified professional care so far. Gradually shifting EOL care from hospitals to community- or home-based settings as desired can avoid late-life hospitalization and maximize scarce resources.^32^ However, traditional family informal care without sufficient professional support is far from meeting the needs of physical comfort and emotional support. Further investigations are needed to investigate whether GHTD meets the preferences and needs of decedents and their families to determine whether EOL care services should aim to facilitate the process of GHTD or to explore the driving forces of GHTD and improve the satisfaction of terminal care.

Our study showed that GHTD was associated with HI schemes. Inequalities in EOL care were growing as illustrated by the widening gap in the proportion of GHTD between URRBMI and UEBMI beneficiaries during the study period. A recently published study reported socioeconomic factors were associated with inequalities in EOL care utilization.^33^ As shown in our data, the demographic and socioeconomic factors were different between URRBMI and UEBMI beneficiaries, but the association of HI schemes with GHTD attenuated and remained significant after adjusted relevant factors, suggesting that the difference in the proportion of GHTD between URRBMI and UEBMI beneficiaries might be partially explained by differences in demographic and socioeconomic factors. For URRBMI beneficiaries, who are already at a socioeconomic disadvantage compared with their UEBMI counterparts, less comprehensive service coverage and lack of financial protections might further aggravate the inequalities in EOL care utilization. Our findings might be helpful for low-and-middle-income countries with similar disparities between HI schemes, especially East and Southeast Asia populations who share a common attitude towards EOL care.^34^ Further investigation is needed to improve the equity of EOL care utilization from the perspective of HI, reimbursement policy in particular.^19^

We found that the proportion of deaths that occurred in the ER or on the way to the hospital showed a declined trend, which might be related to the improved accessibility of medical facilities,^15^ as well as the enhanced awareness of timely medical visit of patients. Meanwhile, an increasing trend was observed for all other places, which might be associated with the increasing mobility of population and urbanization.^35^

Another important implication of our study was the impact of the occurrence of GHTD on the calculation of in-hospital CFR. The corresponding underestimation of CFR would be 48·1% if participants were followed up until 30 days post-hospital discharge. The current administrative reports that used in-hospital CFR for benchmarking purposes need to be interpreted cautiously.^13^ To our knowledge, few clinical studies in China that used the CFR as an endpoint accounted for deaths outside of the hospital.^28^ However, post-discharge outcomes of patients also merit consideration when calculating CFR ^13^, since the occurrence of GHTD is relatively common and grows rapidly in the Chinese population.

Our study has limitations. First, participants with ER or outpatient visits in the last 7 days of life were not included when defining the phenomenon of GHTD because those data were unavailable in the HI system. Thus, our findings may not be generalized to decedents with only ER or outpatient visits. However, the phenomenon of GHTD would be more common if visits to ER or outpatient were taken into account. Second, our study relied on the CKB cohort so that the findings might not be generalizable to the whole Chinese population. Also, EOL preferences of decedents and families were not prospectively collected in CKB. Further exploration would be needed to evaluate the preferences of decedents. Third, data for participants’ HI scheme were only available between 2012 and 2016, therefore, the missing data of the HI scheme for the year of death was the same as the closest scheme available. As the proportion of participants enrolled in the same scheme between 2012 and 2016 remained high (97%), and data from resurveys of CKB participants indicated that only 3% of participants reported being uninsured, it is unlikely that the imputation methods substantially influenced our results. Fourth, in the analysis of GHTD, participants who were uninsured in the year of death were excluded, because the information about hospitalization before death was unavailable. Given the small number of uninsured cases (3·9%), removing them from the analysis does not seem to substantially affect the results. Nonetheless, further studies should pay more attention to this group and the inequities they might experience. Fifth, we did not group the decedents into acute and chronic diseases since differentiation between acute and chronic causes of death based on the underlying cause of death can be difficult in some circumstances. For example, pneumonia is often the direct cause of death ascribed to other common chronic causes, such as lung cancer, Alzheimer's disease, and chronic obstructive lung disease.^36^ Sixth, according to a previous population-based cohort study ^37^, patients with severe mental disorder might experience inequalities in EOL care. However, of the 42,956 participants who died during the study period, only 408 participants’ underlying cause of death was suicide, and the corresponding figure for mental health disorders is 129. This limited sample size precluded us from further stratifying participants by more specific categories of underlying causes of death.

In conclusion, from 2009 to 2017, most of the deaths occurred at home in the CKB cohort. The GHTD phenomenon was common and has been continuously growing during follow-up years. Inequalities in EOL care utilization was still substantial against the background of rapid expansion of hospital beds and also increased hospital admission rates. The development of EOL care services should aim to reduce inequalities and to build a high-quality primary healthcare delivery system and community- or home-based EOL care. Continuous observations of the CKB cohort provide opportunities to evaluate those developments and embed better evidence into policy implementation.

### Contributors

LW and JL conceived and designed the paper. LL, ZC, and JC, as the members of CKB steering committee, designed and supervised the conduct of the whole study, obtained funding, and together with CY, YG, PP, LY, YC, HD, and YL acquired the data. YH and ZS analysed the data. LW and YH drafted the manuscript. LW, JL, YP and BD contributed to the interpretation of the results. JL critically reviewed and revised the manuscript for important intellectual content. All authors reviewed and approved the final manuscript. JL and BD are the guarantors. All authors had access to all data and responsibility for the decision to submit for publication.

### Declaration of Competing Interest

None.

### Acknowledgments

The most important acknowledgment is to the participants in the study and the members of the survey teams in each of the 10 regional centres, as well as to the project-development and -management teams based at Beijing, Oxford and the 10 regional centres. This work was supported by National Natural Science Foundation of China (81941018, 91846303). The CKB baseline survey and the first re-survey were supported by a grant from the Kadoorie Charitable Foundation in Hong Kong. The long-term follow-up is supported by grants (2016YFC0900500) from the National Key R&D Program of China, National Natural Science Foundation of China (81390540), and Chinese Ministry of Science and Technology (2011BAI09B01).

### Data sharing statement

The access policy and procedures are available at www.ckbiobank.org.

## Appendix Supplementary materials

Image, application 1

Image, application 2

Image, application 3
