# Supplementary material for: Place of death and phenomenon of going home to die in Chinese adults: A prospective cohort study
Source: Lancet Reg Health West Pac. 2021 Nov 9;18:100301. doi: 10.1016/j.lanwpc.2021.100301 (PMC8671632; doi:10.1016/j.lanwpc.2021.100301)
Supplement: Supplementary file 1 [file mmc1.docx]

**Table of Contents**

**Page 2:** Members of the China Kadoorie Biobank collaborative group

**Page 3:** Supplementary Methods

**Page 6:** **Table S1.** Characteristics of 42956 CKB participants who died from 2009 to 2017 according to the place of death

**Page 8: Table S2.** Health care before death for the 29828 decedents who died at home according to the health insurance schemes

**Page 9: Table S3.** Characteristics of decedents who received inpatient care within the last 3, 7, 14, or 30 days of life Members of the China Kadoorie Biobank collaborative group

**Page 11: Table S4.** The extent to which in-hospital case fatality rates were underestimated when taking decedents GHTD from the hospital into account

**Page 12:** **Table S5.** PRs (95% CIs) for the likelihood of GHTD from the hospital associated with health insurance schemes among decedents who received inpatient care in the last 3, 14, or 30 days of life

**Page 14: Table S6.** PRs (95% CIs) for the likelihood of GHTD from the hospital associated with health insurance schemes by the underlying cause of death among 13777 decedents who received inpatient care in the last 7 days of life

**Page 15: Fig. S1.** Changes in the proportion of place of death from 2009 to 2017 by health insurance schemes

**Page 16: Fig. S2.** Changes in the proportion of going home to die from 2009 to 2017 among decedents who received inpatient care within the last 3, 14, or 30 days of life

**Members of the China Kadoorie Biobank collaborative group**

**International Steering Committee:** Junshi Chen, Zhengming Chen (PI), Robert Clarke, Rory Collins, Yu Guo, Liming Li (PI), Jun Lv, Richard Peto, Robin Walters. **International Co-ordinating Centre, Oxford:** Daniel Avery, Ruth Boxall, Derrick Bennett, Yumei Chang, Yiping Chen, Zhengming Chen, Robert Clarke, Huaidong Du, Simon Gilbert, Alex Hacker, Mike Hill, Michael Holmes, Andri Iona, Christiana Kartsonaki, Rene Kerosi, Ling Kong, Om Kurmi, Garry Lancaster, Sarah Lewington, Kuang Lin, John McDonnell, Iona Millwood, Qunhua Nie, Jayakrishnan Radhakrishnan, Paul Ryder, Sam Sansome, Dan Schmidt, Paul Sherliker, Rajani Sohoni, Becky Stevens, Iain Turnbull, Robin Walters, Jenny Wang, Lin Wang, Neil Wright, Ling Yang, Xiaoming Yang. **National Co-ordinating Centre, Beijing:** Yu Guo, Xiao Han, Can Hou, Jun Lv, Pei Pei, Chao Liu, Canqing Yu. **10 Regional Co-ordinating Centres: Qingdao CDC:** Zengchang Pang, Ruqin Gao, Shanpeng Li, Shaojie Wang, Yongmei Liu, Ranran Du, Yajing Zang, Liang Cheng, Xiaocao Tian, Hua Zhang, Yaoming Zhai, Feng Ning, Xiaohui Sun, Feifei Li. **Licang CDC:** Silu Lv, Junzheng Wang, Wei Hou. **Heilongjiang Provincial CDC:** Mingyuan Zeng, Ge Jiang, Xue Zhou. **Nangang CDC:** Liqiu Yang, Hui He, Bo Yu, Yanjie Li, Qinai Xu,Quan Kang, Ziyan Guo. **Hainan Provincial CDC:** Dan Wang, Ximin Hu, Jinyan Chen, Yan Fu, Zhenwang Fu, Xiaohuan Wang. **Meilan CDC:** Min Weng, Zhendong Guo, Shukuan Wu,Yilei Li, Huimei Li, Zhifang Fu. **Jiangsu Provincial CDC:** Ming Wu, Yonglin Zhou, Jinyi Zhou, Ran Tao, Jie Yang, Jian Su. **Suzhou CDC:** Fang liu, Jun Zhang, Yihe Hu, Yan Lu, , Liangcai Ma, Aiyu Tang, Shuo Zhang, Jianrong Jin, Jingchao Liu. **Guangxi Provincial CDC:** Zhenzhu Tang, Naying Chen, Ying Huang. **Liuzhou CDC:** Mingqiang Li, Jinhuai Meng, Rong Pan, Qilian Jiang, Jian Lan,Yun Liu, Liuping Wei, Liyuan Zhou, Ningyu Chen Ping Wang, Fanwen Meng, Yulu Qin,, Sisi Wang. **Sichuan Provincial CDC:** Xianping Wu, Ningmei Zhang, Xiaofang Chen,Weiwei Zhou. **Pengzhou CDC:** Guojin Luo, Jianguo Li, Xiaofang Chen, Xunfu Zhong, Jiaqiu Liu, Qiang Sun. **Gansu Provincial CDC:** Pengfei Ge, Xiaolan Ren, Caixia Dong. **Maiji CDC:** Hui Zhang, Enke Mao, Xiaoping Wang, Tao Wang, Xi zhang. **Henan Provincial CDC:** Ding Zhang, Gang Zhou, Shixian Feng, Liang Chang, Lei Fan. **Huixian CDC:** Yulian Gao, Tianyou He, Huarong Sun, Pan He, Chen Hu, Xukui Zhang, Huifang Wu, Pan He. **Zhejiang Provincial CDC:** Min Yu, Ruying Hu, Hao Wang. Tongxiang CDC: Yijian Qian, Chunmei Wang, Kaixu Xie, Lingli Chen, Yidan Zhang, Dongxia Pan, Qijun Gu. **Hunan Provincial CDC:** Yuelong Huang, Biyun Chen, Li Yin, Huilin Liu, Zhongxi Fu, Qiaohua Xu. **Liuyang CDC:** Xin Xu, Hao Zhang, Huajun Long, Xianzhi Li, Libo Zhang, Zhe Qiu.

**Supplementary Methods**

***Baseline questionnaires***

Before enrolment, participants were informed of the details of this study and gave a written informed consent, which allows access to their medical records and long-term storage of blood for anonymized and non-specified medical research purposes. All participants completed an interviewer-administered questionnaire, underwent a range of physical measurements at baseline. Information collected at baseline was entered into a laptop-based direct data entry system, with built-in functions to avoid missing items and to prevent logic errors during the interview. Serial resurveys, the 1^st^ resurvey in 2008, and the 2^nd^ resurvey during 2012-2014, in 5% of the study population indicated a high level of agreement for replicate measurements for questionnaire data.

***Follow-up data***

The vital status of study participants in the CKB study was ascertained through official Disease Surveillance Points (DSP) death registries (Yang G et al., 1997), which covers all study areas. To maximize the death ascertainment of participants and identify participants who had moved permanently out of the study areas, annual checks with local residential records, health insurance (HI) records, and active confirmation of survival were conducted by local residential administrators (Chen Z et al., 2011). The DSP system provides reliable and completed registration for causes of death for almost all adults (Yang G et al., 2005). For the few deaths (<5%) that occurred without medical attention, a standardized verbal autopsy was used to determine probable causes of death according to the symptoms or signs described by informants (usually family members) (Yang G et al., 2006).

The hospitalization records before death were obtained through linkages to the local HI system, which was renewed annually. Based on the initial linkage established in study areas, personal data have been matched to the HI system for almost all participants using unique personal identification numbers. Both urban and rural participants had similar proportions of successful linkage to HI databases.

***Description of different types of HI schemes in China***

(1) Urban Employee Basic Medical Insurance (UEBMI): mandatory scheme for urban employees. This scheme is funded by both employer (8-10%) and employee (2%) contributions. Retired individuals who were previously employed and covered by UEBMI remain enrolled in UEBMI.

(2) Urban Resident Basic Medical Insurance (URBMI): voluntary scheme for children, students, urban residents without formal employment, and elderly without previous employment. This scheme is mainly funded by government subsidies (~70% of the total funds).

(3) New Rural Cooperative Medical Scheme (NRCMS): voluntary scheme for rural residents. This scheme is mainly funded by government subsidies (~70% of the total funds).

(4) Urban and Rural Residents’ Basic Medical Insurance (URRBMI): To provide better coverage services and financial protection for the rural residents and to reduce the healthcare utilization inequity in rural areas, China began to establish the URRBMI scheme based on the URBMI and NRCMS. In four of the ten CKB study areas, URBMI and NRCMS were also merged to form the URRBMI scheme from 2012-2013 onwards.

***Imputation methods used for missing HI scheme***

The imputation methods of the HI scheme we used were complying with a previous CKB study (Levy et al. 2020), with minor adaptations. Participants were linked to individual HI schemes annually since 2012, using the participants’ unique personal identification numbers. Information on uninsured participants was also provided.

Given the study period was limited to the interval between 2009 and 2017, data for participants’ HI schemes for the period 2009-2011 and year 2017 were imputed based on the closest scheme available between 2012 and 2016. In China, individuals are enrolled in a particular HI scheme mainly depending on their employment status. Furthermore, at entry into the study (2004-2008), 82% of participants self-reported being insured. This proportion increased to 97% and 98% in the 5% sample of individuals who participated in the 1^st^ resurvey (2008) and the 2^nd^ resurvey (2013-2014), respectively. Therefore, it was unlikely for participants to be uninsured. Uninsured participants were excluded from analyses by HI type, due to the small number of cases.

In the imputation for HI schemes, an assumption was made that middle-aged and older individuals in China such as participants in CKB (ie, mean age 51.5 years at baseline) were unlikely to change employment and, hence, their HI scheme. The imputation of the HI scheme before death for participants who died in 2009-2017 was performed as outlined below:

(1) The "unknown" HI scheme was used to code participants that were insured, but whose HI scheme could not be identified. To reduce the proportion of unknown HI schemes in 2012-2016, we first imputed the unknown scheme using the HI scheme that participants were enrolled in during the previous year (if not uninsured or unknown), and then using the HI scheme they were enrolled in during the following year (if not uninsured or unknown).

(2) Between 2009 and 2011, all participants were assumed to be enrolled in the same HI scheme as in 2012. For participants still alive until 2017, their HI scheme was assumed to be the same as in 2016.

(3) Participants who died before 2012 were assumed to be insured and their HI type was replaced by a "Missing" scheme.

(4) For participants uninsured during 2009-17 (the mean proportion was 7.1%), we checked whether they had any admission reported in HI records for the corresponding year. If they had an admission, we coded them as having an unknown HI type for that year.

(5) Participants with a missing or unknown HI scheme in 2009-2017, had their HI scheme replaced by one of the two main schemes (UEBMI, URRBMI [formed by URBMI and NRCMS]) based on their self-reported occupation at entry into the study:

a) For participants that reported being agriculture or related workers, we replaced their missing/unknown scheme with URRBMI.

b) For participants living in rural areas, we replaced their missing/unknown scheme with URRBMI, as the majority of individuals living in rural areas were enrolled in NRCMS in 2012-2016.

c) For participants living in urban areas and in formal employment (factory worker, sales and services, administration/manager, professional/technical), their missing scheme was replaced by UEBMI.

d) For participants living in urban areas and in informal employment (housewife/househusband, unemployed, other or non-stated), we replaced their missing/unknown scheme with URRBMI.

e) For retired individuals living in urban areas, if they were male and aged ≥60 or female and aged ≥55 (official retirement ages), their missing/unknown scheme was replaced by UEBMI, as the majority of retired individuals in urban areas were enrolled in UEBMI in 2012-2016.

f) Remaining participants with an unknown/missing information about the scheme (<1% in 2009-2017) were combined with the uninsured category.

(6) In this study, the HI scheme we used mainly was the scheme that participants enrolled in for the year of death. To further reduce the proportion of uninsured HI types for the year of death, the uninsured scheme for death year was imputed using the HI scheme they were enrolled in during the year before death (if not uninsured).

**Table S1. Characteristics of 42956 CKB participants who died from 2009 to 2017 according to the place of death**

|  | **Overall** | **Place of death** | | | |
| --- | --- | --- | --- | --- | --- |
|  |  | **Home** | **Hospital** | **Emergency room or on the way to the hospital** | **All other places** |
| **Decedents ^a^, n (%)** | 42956 (100.0) | 30719 (71.5) | 9260 (21.6) | 864 (2.0) | 2113 (4.9) |
| **Age at death, year (SD)** | 69.3 (10.0) | 69.5 (9.7) | 69.5 (10.1) | 65.1 (11.2) | 66.3 (11.7) |
| **Sex, n (%)** |  |  |  |  |  |
| Male | 24171 (56.3) | 17167 (55.9) | 5246 (56.7) | 514 (59.5) | 1244 (58.9) |
| Female | 18785 (43.7) | 13552 (44.1) | 4014 (43.3) | 350 (40.5) | 869 (41.1) |
| **Place of residence, n (%)** |  |  |  |  |  |
| Rural area | 26171 (60.9) | 23656 (77.0) | 1344 (14.5) | 300 (34.7) | 871 (41.2) |
| Urban area | 16785 (39.1) | 7063 (23.0) | 7916 (85.5) | 564 (65.3) | 1242 (58.8) |
| **Education level, n (%)** |  |  |  |  |  |
| No formal school | 12823 (29.9) | 11063 (36.0) | 1187 (12.8) | 165 (19.1) | 408 (19.3) |
| Primary and junior high school | 24857 (57.9) | 17527 (57.1) | 5525 (59.7) | 541 (62.6) | 1264 (59.8) |
| Senior high school and above | 5276 (12.3) | 2129 (6.9) | 2548 (27.5) | 158 (18.3) | 441 (20.9) |
| **Marital status, n (%)** |  |  |  |  |  |
| Married | 34560 (80.5) | 24442 (79.6) | 7672 (82.9) | 732 (84.7) | 1714 (81.1) |
| Others **^b^** | 8396 (19.5) | 6277 (20.4) | 1588 (17.1) | 132 (15.3) | 399 (18.9) |
| **Annual household income (RMB yuan), n (%)** | |  |  |  |  |
| <10000 | 17473 (40.7) | 14625 (47.6) | 1869 (20.2) | 254 (29.4) | 725 (34.3) |
| 10000-19999 | 12620 (29.4) | 8196 (26.7) | 3420 (36.9) | 309 (35.8) | 695 (32.9) |
| 20000-34999 | 8041 (18.7) | 4866 (15.8) | 2543 (27.5) | 193 (22.3) | 439 (20.8) |
| ≥35000 | 4822 (11.2) | 3032 (9.9) | 1428 (15.4) | 108 (12.5) | 254 (12.0) |
| **Occupation, n (%)** |  |  |  |  |  |
| Managers or professionals | 626 (1.5) | 247 (0.8) | 313 (3.4) | 8 (0.9) | 58 (2.7) |
| Agricultural, manufacturing, services or sales workers | 21016 (48.9) | 17791 (57.9) | 1971 (21.3) | 339 (39.2) | 915 (43.3) |
| Other occupations, housework, retired, or unemployed | 21314 (49.6) | 12681 (41.3) | 6976 (75.3) | 517 (59.8) | 1140 (54.0) |
| **Health insurance scheme ^c^, n (%)** |  |  |  |  |  |
| UEBMI | 12985 (30.2) | 4978 (16.2) | 6783 (73.3) | 437 (50.6) | 787 (37.2) |
| URRBMI | 28292 (65.9) | 24850 (80.9) | 2077 (22.4) | 378 (43.8) | 987 (46.7) |
| Uninsured | 1679 (3.9) | 891 (2.9) | 400 (4.3) | 49 (5.7) | 339 (16.0) |
| **Household size, n (%)** |  |  |  |  |  |
| Live alone | 3054 (7.1) | 2092 (6.8) | 721 (7.8) | 60 (6.9) | 181 (8.6) |
| 2 people | 12618 (29.4) | 8129 (26.5) | 3596 (38.8) | 304 (35.2) | 589 (27.9) |
| 3-4 people | 12597 (29.3) | 8068 (26.3) | 3426 (37.0) | 315 (36.5) | 788 (37.3) |
| ≥5 people | 14687 (34.2) | 12430 (40.5) | 1517 (16.4) | 185 (21.4) | 555 (26.3) |
| **Underlying cause of death, n (%)** |  |  |  |  |  |
| Malignant neoplasms | 13546 (31.5) | 9418 (30.7) | 3695 (39.9) | 153 (17.7) | 280 (13.3) |
| Ischemic heart diseases | 6727 (15.7) | 4798 (15.6) | 1372 (14.8) | 263 (30.4) | 294 (13.9) |
| Cerebrovascular diseases | 9656 (22.5) | 7696 (25.1) | 1576 (17.0) | 132 (15.3) | 252 (11.9) |
| Diseases of the respiratory system | 4046 (9.4) | 3039 (9.9) | 903 (9.8) | 43 (5.0) | 61 (2.9) |
| External causes | 2635 (6.1) | 1495 (4.9) | 382 (4.1) | 164 (19.0) | 594 (28.1) |
| Other causes | 6346 (14.8) | 4273 (13.9) | 1332 (14.4) | 109 (12.6) | 632 (29.9) |

Abbreviations: CKB, China Kadoorie Biobank; SD, standard deviation; UEBMI: Urban Employee Basic Medical Insurance; URRBMI: Urban and Rural Residents’ Basic Medical Insurance.

Column percentages were provided unless indicated otherwise.

^a^ Row percentages were provided.

^b^ Including widowed, divorced or separated, or never married.

^c^ Information on health insurance scheme is for the year of death.

Table S2. Health care before death for the 29828 decedents who died at home according to the health insurance schemes

|  | **Overall (n=29828)** | **Health insurance schemes ^a^** | | ***P*-value ^b^** | |
| --- | --- | --- | --- | --- | --- |
|  |  | **UEBMI (n=4978)** | **URRBMI (n=24850)** |  |  |
| **Hospitalization prior death, n (%)** |  |  |  |  | |
| Hospitalization from 2009 to the date of death | 20519 (68.8) | 4010 (80.6) | 16509 (66.4) | <0.001 | |
| Hospitalization during last 2 years of life | 18823 (63.1) | 3640 (73.1) | 15183 (61.1) | | <0.001 |
| Hospitalization during last 1 year of life | 16504 (55.3) | 3231 (64.9) | 13273 (53.4) | <0.001 | |
| Hospitalization during last 3 months of life | 11600 (38.9) | 2231 (44.8) | 9369 (37.7) | <0.001 | |
| Hospitalization during last 30 days of life | 8209 (27.5) | 1517 (30.5) | 6692 (26.9) | <0.001 | |
| Hospitalization during last 14 days of life | 6281 (21.1) | 1144 (23.0) | 5137 (20.7) | <0.001 | |
| Hospitalization during last 7 days of life | 4917 (16.5) | 888 (17.8) | 4029 (16.2) | 0.005 | |
| Hospitalization during last 3 days of life | 3751 (12.6) | 671 (13.5) | 3080 (12.4) | 0.035 | |
| **The length of stay of the last hospitalization before death, median (IQR) ^c^** | 9.0 (5.0, 15.0) | 11.0 (6.0, 17.0) | 8.0 (5.0, 14.0) | <0.001 | |
| **Days from the date of last hospital discharge to the date of death, median (IQR) ^d^** | 4.0 (0.0, 14.0) | 4.0 (0.0, 14.0) | 4.0 (0.0, 14.0) | 0.791 | |

Abbreviations: UEBMI: Urban Employee Basic Medical Insurance; URRBMI: Urban and Rural Residents’ Basic Medical Insurance; IQR: interquartile range.

^a^ Information on health insurance scheme is for the year of death.

^b^ The Chi-Squared test was used for categorical variables and the Wilcoxon rank-sum test was used for continuous variables.

^c^ Decedents without hospitalization records during follow-up (n=8098), and decedents whose discharge date of the last hospitalization before death were implausible or missing (n=1320) were excluded.

^d^ For 8209 decedents discharged from the hospital within 30 days before death at home.

Table S3. Characteristics of decedents who received inpatient care within the last 3, 7, 14, or 30 days of life

|  | **Die in the hospital (n=8860)** | **Different definitions of GHTD** | | | |
| --- | --- | --- | --- | --- | --- |
|  |  | **GHTD: 3 days (n=3751)** | **GHTD: 7 days (n=4917)** | **GHTD: 14 days (n=6281)** | **GHTD: 30 days (n=8209)** |
| **Age at death, year (SD)** | 69.6 (10.0) | 68.9 (9.5) | 69.0 (9.5) | 69.2 (9.5) | 69.3 (9.4) |
| **Sex, n (%)** |  |  |  |  |  |
| Male | 5016 (56.6) | 2182 (58.2) | 2849 (57.9) | 3641 (58.0) | 4740 (57.7) |
| Female | 3844 (43.4) | 1569 (41.8) | 2068 (42.1) | 2640 (42.0) | 3469 (42.3) |
| **Place of residence, n (%)** |  |  |  |  |  |
| Rural area | 1321 (14.9) | 2883 (76.9) | 3749 (76.2) | 4757 (75.7) | 6159 (75.0) |
| Urban area | 7539 (85.1) | 868 (23.1) | 1168 (23.8) | 1524 (24.3) | 2050 (25.0) |
| **Education level, n (%)** |  |  |  |  |  |
| No formal school | 1148 (13.0) | 1218 (32.5) | 1617 (32.9) | 2065 (32.9) | 2727 (33.2) |
| Primary and junior high school | 5270 (59.5) | 2336 (62.3) | 3025 (61.5) | 3858 (61.4) | 4983 (60.7) |
| Senior high school and above | 2442 (27.6) | 197 (5.3) | 275 (5.6) | 358 (5.7) | 499 (6.1) |
| **Marital status, n (%)** |  |  |  |  |  |
| Married | 1527 (17.2) | 592 (15.8) | 753 (15.3) | 1000 (15.9) | 1358 (16.5) |
| Others **^a^** | 7333 (82.8) | 3159 (84.2) | 4164 (84.7) | 5281 (84.1) | 6851 (83.5) |
| **Annual household income (RMB yuan), n (%)** |  |  |  |  |  |
| <10000 | 1801 (20.3) | 1461 (38.9) | 1952 (39.7) | 2508 (39.9) | 3326 (40.5) |
| 10000-19999 | 3282 (37.0) | 926 (24.7) | 1244 (25.3) | 1626 (25.9) | 2129 (25.9) |
| 20000-34999 | 2423 (27.3) | 779 (20.8) | 997 (20.3) | 1256 (20.0) | 1612 (19.6) |
| ≥35000 | 1354 (15.3) | 585 (15.6) | 724 (14.7) | 891 (14.2) | 1142 (13.9) |
| **Occupation, n (%)** |  |  |  |  |  |
| Managers or professionals | 309 (3.5) | 28 (0.7) | 35 (0.7) | 47 (0.7) | 61 (0.7) |
| Agricultural, manufacturing, services or sales workers | 1942 (21.9) | 2417 (64.4) | 3129 (63.6) | 3948 (62.9) | 5114 (62.3) |
| Other occupations, housework, retired, or unemployed | 6609 (74.6) | 1306 (34.8) | 1753 (35.7) | 2286 (36.4) | 3034 (37.0) |
| **Health insurance scheme ^b^, n (%)** |  |  |  |  |  |
| UEBMI | 6783 (76.6) | 671 (17.9) | 888 (18.1) | 1144 (18.2) | 1517 (18.5) |
| URRBMI | 2077 (23.4) | 3080 (82.1) | 4029 (81.9) | 5137 (81.8) | 6692 (81.5) |
| **Household size, n (%)** |  |  |  |  |  |
| Live alone | 699 (7.9) | 218 (5.8) | 282 (5.7) | 373 (5.9) | 503 (6.1) |
| 2 people | 3454 (39.0) | 986 (26.3) | 1308 (26.6) | 1705 (27.1) | 2265 (27.6) |
| 3-4 people | 3251 (36.7) | 997 (26.6) | 1299 (26.4) | 1649 (26.3) | 2135 (26.0) |
| ≥5 people | 1456 (16.4) | 1550 (41.3) | 2028 (41.2) | 2554 (40.7) | 3306 (40.3) |
| **Underlying cause of death, n (%)** |  |  |  |  |  |
| Malignant neoplasms | 3513 (39.7) | 1462 (39.0) | 1945 (39.6) | 2581 (41.1) | 3500 (42.6) |
| Ischemic heart diseases | 1331 (15.0) | 404 (10.8) | 545 (11.1) | 697 (11.1) | 907 (11.0) |
| Cerebrovascular diseases | 1497 (16.9) | 893 (23.8) | 1112 (22.6) | 1344 (21.4) | 1646 (20.1) |
| Diseases of the respiratory system | 873 (9.9) | 429 (11.4) | 572 (11.6) | 718 (11.4) | 914 (11.1) |
| External causes | 369 (4.2) | 87 (2.3) | 101 (2.1) | 125 (2.0) | 169 (2.1) |
| Other causes | 1277 (14.4) | 476 (12.7) | 642 (13.1) | 816 (13.0) | 1073 (13.1) |

Abbreviations: GHTD, going home to die; SD, standard deviation; UEBMI: Urban Employee Basic Medical Insurance; URRBMI: Urban and Rural Residents’ Basic Medical Insurance.

Column percentages were provided.

^a^ Including widowed, divorced or separated, or never married.

^b^ Information on health insurance scheme is for the year of death.

**Table S4. The extent to which in-hospital case fatality rates were underestimated when taking decedents GHTD from the hospital into account**

|  | **Different definitions of GHTD** | | | |
| --- | --- | --- | --- | --- |
|  | **Discharged from the hospital to die at home within 3 days** | **Discharged from the hospital to die at home within 7 days** | **Discharged from the hospital to die at home within 14 days** | **Discharged from the hospital to die at home within 30 days** |
| The number of decedents who died at home and met the definition of GHTD during 2009-2017 ($n_{1}$) | 3751 | 4917 | 6281 | 8209 |
| Corresponding degree of underestimation (%) ^a^ | 29.7 | 35.7 | 41.5 | 48.1 |

Abbreviations: GHTD, going home to die.

In-hospital case fatality rate (CFR) was calculated based on status at discharge, by dividing the number of deaths during the hospital stay by the total number of hospital discharges. The number of decedents who died in the hospital during 2009-2017 ($n_{2}$) was 8860.

^a^ Assuming that the number of hospital discharges (denominator) of cohort participants during 2009-2017 was N, the in-hospital CFR_1_ that commonly used in clinical scenarios can be calculated as $\frac{n_{2}}{N}.$ However, since some participants chose to discharge from the hospital to die at home or were discharged against medical advice, the in-hospital CFR_2_ that is in line with actual situation should be calculated as $\frac{n_{1}+n_{2}}{N}$. The degree of underestimation is equal to $\frac{n_{1}}{n_{1}+n_{2}}$.

**Table S5. PRs (95% CIs) for the likelihood of GHTD from the hospital associated with health insurance schemes among decedents who received inpatient care in the last 3, 14, or 30 days of life**

|  | **Overall** | |  | **Urban areas** | |  | **Rural areas** | |
| --- | --- | --- | --- | --- | --- | --- | --- | --- |
|  | **UEBMI** | **URRBMI** |  | **UEBMI** | **URRBMI** |  | **UEBMI** | **URRBMI** |
| **Decedents received inpatient care in the last 3 days (n=12611)** | | | | | | | | |
| **Decedents/GHTD, n/n** | 7454/671 | 5157/3080 |  | 7067/449 | 1340/419 |  | 387/222 | 3817/2661 |
| **GHTD, %** | 9.0 | 59.7 |  | 6.4 | 31.3 |  | 57.4 | 69.7 |
| **Without adjustment** |  |  |  |  |  |  |  |  |
| PRs (95% CIs) | 1.00 | 6.63 (6.15, 7.16) |  | 1.00 | 4.92 (4.37, 5.55) |  | 1.00 | 1.22 (1.11, 1.33) |
| *P*-value | <0.001 | |  | <0.001 | |  | <0.001 | |
| **Model 1** |  |  |  |  |  |  |  |  |
| PRs (95% CIs) | 1.00 | 1.30 (1.21, 1.40) |  | 1.00 | 1.37 (1.22, 1.53) |  | 1.00 | 1.24 (1.14, 1.36) |
| *P*-value | <0.001 | |  | <0.001 | |  | <0.001 | |
| **Model 2** |  |  |  |  |  |  |  |  |
| PRs (95% CIs) | 1.00 | 1.19 (1.11, 1.28) |  | 1.00 | 1.19 (1.05, 1.34) |  | 1.00 | 1.13 (1.03, 1.24) |
| *P*-value | <0.001 | |  | 0.005 | |  | 0.007 | |
| **Model 3 ^a^** |  |  |  |  |  |  |  |  |
| PRs (95% CIs) | 1.00 | 1.22 (1.14, 1.31) |  | 1.00 | 1.15 (1.02, 1.30) |  | 1.00 | 1.25 (1.14, 1.36) |
| *P*-value | <0.001 | |  | 0.025 | |  | <0.001 | |
| **Decedents received inpatient care in the last 14 days (n=15141)** | | | | | | | | |
| **Decedents/GHTD** | 7927/1144 | 7214/5137 |  | 7437/819 | 1626/705 |  | 490/325 | 5588/4432 |
| **GHTD, %** | 14.4 | 71.2 |  | 11.0 | 43.4 |  | 66.3 | 79.3 |
| **Without adjustment** |  |  |  |  |  |  |  |  |
| PRs (95% CIs) | 1.00 | 4.93 (4.67, 5.22) |  | 1.00 | 3.94 (3.62, 4.29) |  | 1.00 | 1.20 (1.12, 1.28) |
| *P*-value | <0.001 | |  | <0.001 | |  | <0.001 | |
| **Model 1** |  |  |  |  |  |  |  |  |
| PRs (95% CIs) | 1.00 | 1.27 (1.20, 1.34) |  | 1.00 | 1.32 (1.22, 1.44) |  | 1.00 | 1.21 (1.13, 1.29) |
| *P*-value | <0.001 | |  | <0.001 | |  | <0.001 | |
| **Model 2** |  |  |  |  |  |  |  |  |
| PRs (95% CIs) | 1.00 | 1.18 (1.11, 1.24) |  | 1.00 | 1.17 (1.07, 1.28) |  | 1.00 | 1.13 (1.06, 1.21) |
| *P*-value | <0.001 | |  | 0.001 | |  |  | <0.001 |
| **Model 3 ^a^** |  |  |  |  |  |  |  |  |
| PRs (95% CIs) | 1.00 | 1.19 (1.13, 1.26) |  | 1.00 | 1.15 (1.05, 1.26) |  | 1.00 | 1.20 (1.12, 1.28) |
| *P*-value | <0.001 | |  | 0.003 | |  | <0.001 | |
| **Decedents received inpatient care in the last 30 days (n=17069)** | | | | | | | | |
| **Decedents/GHTD** | 8300/1517 | 8769/6692 |  | 7734/1116 | 1855/934 |  | 566/401 | 6914/5758 |
| **GHTD, %** | 18.3 | 76.3 |  | 14.4 | 50.4 |  | 70.8 | 83.3 |
| **Without adjustment** |  |  |  |  |  |  |  |  |
| PRs (95% CIs) | 1.00 | 4.18 (3.98, 4.38) |  | 1.00 | 3.49 (3.25, 3.74) |  | 1.00 | 1.18 (1.11, 1.24) |
| *P*-value | <0.001 | |  | <0.001 | |  | <0.001 | |
| **Model 1** |  |  |  |  |  |  |  |  |
| PRs (95% CIs) | 1.00 | 1.25 (1.19, 1.31) |  | 1.00 | 1.31 (1.22, 1.40) |  | 1.00 | 1.18 (1.12, 1.25) |
| *P*-value | <0.001 | |  | <0.001 | |  | <0.001 | |
| **Model 2** |  |  |  |  |  |  |  |  |
| PRs (95% CIs) | 1.00 | 1.16 (1.11, 1.22) |  | 1.00 | 1.16 (1.08, 1.26) |  | 1.00 | 1.12 (1.06, 1.18) |
| *P*-value | <0.001 | |  | <0.001 | |  | <0.001 | |
| **Model 3 ^a^** |  |  |  |  |  |  |  |  |
| PRs (95% CIs) | 1.00 | 1.18 (1.13, 1.23) |  | 1.00 | 1.15 (1.07, 1.25) |  | 1.00 | 1.17 (1.11, 1.23) |
| *P*-value | <0.001 | |  | <0.001 | |  | <0.001 | |

Abbreviations: PR, prevalence ratio; CI, confidence interval; GHTD, going home to die; UEBMI: Urban Employee Basic Medical Insurance; URRBMI: Urban and Rural Residents’ Basic Medical Insurance.

Information on health insurance scheme is for the year of death. The modified Poisson models were used. Model 1 was adjusted for age at death (years), sex (male or female), and ten study areas; model 2 was further adjusted for marital status (married or others), household income (<10000, 10000-19999, 20000-34999, or ≥35000 RMB yuan), education attainment (no formal school, primary and junior high school, or senior high school and above), household size including self (1, 2, 3-4, or ≥5), occupation (managers or professionals, agricultural, manufacturing, services or sales workers, other occupations, housework, retired, or unemployed); model 3 was adjusted for model 2 plus the underlying cause of death, and year of death.

^a^ The interaction p-values for statistical interaction between the place of residence (urban and rural areas) and the health insurance scheme were 0.958, 0.602, and 0.347 when the alternative intervals used to define the GHTD phenomenon were 3, 14, and 30 days, respectively.

**Table S6. PRs (95% CIs) for the likelihood of GHTD from the hospital associated with health insurance schemes by the underlying cause of death among 13777 decedents who received inpatient care in the last 7 days of life**

|  | **UEBMI** | **URRBMI** |
| --- | --- | --- |
|  |  |  |
| **Malignant neoplasms (n=5458)** | | |
| Decedents/GHTD, n/n | 3353/411 | 2105/1534 |
| GHTD, % | 12.3 | 72.9 |
| PRs (95% CIs) | 1.00 | 1.22 (1.13, 1.31) |
| *P*-value | <0.001 | |
| **Ischemic heart diseases (n=1876)** | | |
| Decedents/GHTD, n/n | 1102/122 | 774/423 |
| GHTD, % | 11.1 | 54.7 |
| PRs (95% CIs) | 1.00 | 1.16 (0.90, 1.50) |
| *P*-value | 0.256 | |
| **Cerebrovascular diseases (n=2609)** | | |
| Decedents/GHTD, n/n | 1251/164 | 1358/948 |
| GHTD, % | 13.1 | 69.8 |
| PRs (95% CIs) | 1.00 | 1.04 (0.92, 1.18) |
| *P*-value | 0.520 | |
| **Diseases of the respiratory system (n=1445)** | | |
| Decedents/GHTD, n/n | 741/60 | 704/512 |
| GHTD, % | 8.1 | 72.7 |
| PRs (95% CIs) | 1.00 | 1.25 (1.01, 1.54) |
| *P*-value | 0.039 | |
| **External causes (n=470)** | | |
| Decedents/GHTD, n/n | 167/14 | 303/87 |
| GHTD, % | 8.4 | 28.7 |
| PRs (95% CIs) | 1.00 | 1.21 (0.69, 2.12) |
| *P*-value | 0.497 | |
| **Other causes (n=1919)** | | |
| Decedents/GHTD, n/n | 1057/117 | 862/525 |
| GHTD, % | 11.1 | 60.9 |
| PRs (95% CIs) | 1.00 | 1.35 (1.11, 1.64) |
| *P*-value | 0.003 | |

Abbreviations: PR, prevalence ratio; CI, confidence interval; GHTD, going home to die; UEBMI: Urban Employee Basic Medical Insurance; URRBMI: Urban and Rural Residents’ Basic Medical Insurance.

Information on health insurance scheme is for the year of death. The modified Poisson models were used. Covariate adjusted for in the model included all of the variables in model 3 of Table 2, except for the underlying cause of death.

**
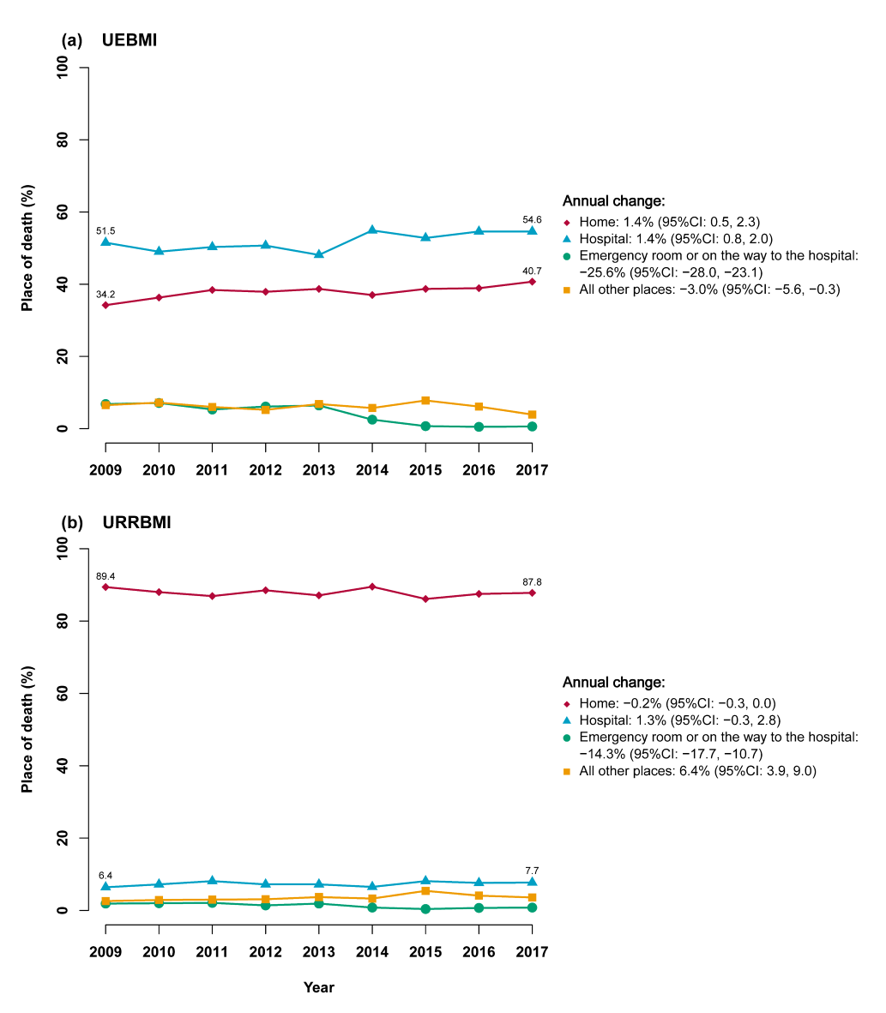
**

**Fig. S1. Changes in the proportion of place of death from 2009 to 2017 by health insurance schemes**

Abbreviations: UEBMI: Urban Employee Basic Medical Insurance; URRBMI: Urban and Rural Residents’ Basic Medical Insurance.

Information on health insurance scheme is for the year of death, and decedents who were uninsured were excluded due to the small number of cases. The Poisson models were adjusted for age at death, sex, and study area.


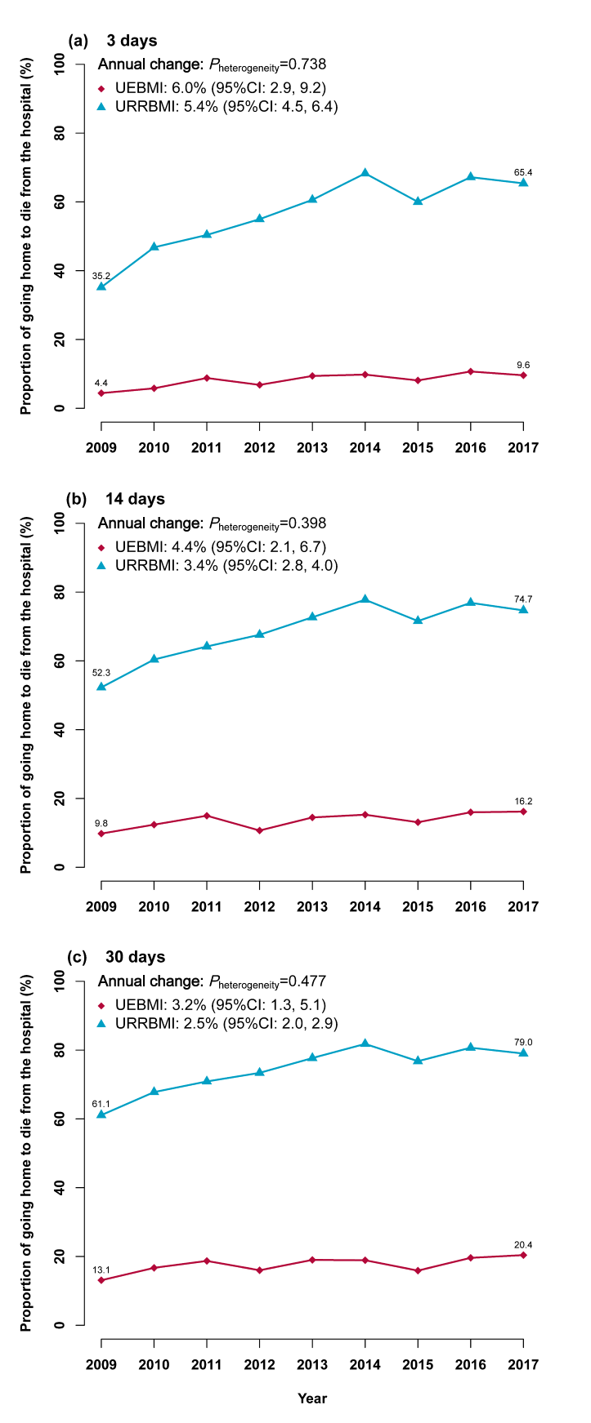


**Fig. S2. Changes in the proportion of going home to die from 2009 to 2017 among decedents who received inpatient care within the last 3, 14, or 30 days of life**

Abbreviations: UEBMI: Urban Employee Basic Medical Insurance; URRBMI: Urban and Rural Residents’ Basic Medical Insurance.

The Poisson models were adjusted for age at death, sex, and study area. Three different definitions of “going home to die from the hospital” were applied: discharged from the hospital to die at home within 3 days (panel a), discharged from the hospital to die at home within 14 days (panel b), and discharged from the hospital to die at home within 30 days (panel c).
