# Supplementary material for: Place of death and phenomenon of going home to die in Chinese adults: A prospective cohort study
Source: Lancet Reg Health West Pac. 2021 Nov 9;18:100301. doi: 10.1016/j.lanwpc.2021.100301 (PMC8671632; doi:10.1016/j.lanwpc.2021.100301)
Supplement: Supplementary file 2 [file mmc2.docx]

**Caption for supplementary material**

Translated Abstract

Supplemental Appendix
