# Supplementary material for: Place of death and phenomenon of going home to die in Chinese adults: A prospective cohort study
Source: Lancet Reg Health West Pac. 2021 Nov 9;18:100301. doi: 10.1016/j.lanwpc.2021.100301 (PMC8671632; doi:10.1016/j.lanwpc.2021.100301)
Supplement: Supplementary file 3 [file mmc3.docx]

**This translation in Chinese was submitted by the authors and we reproduce it as supplied. It has not been peer reviewed. Our editorial processes have only been applied to the original abstract in English, which should serve as reference for this manuscript.**

**摘要**

**背景：**中国人口正在快速老龄化，却没有可持续的临终医疗服务。在这种背景下，中国人群的死亡地点和回家死亡的现象仍待研究。

**方法：**中国慢性病前瞻性研究（China Kadoorie Biobank）纳入了来自全国10个地区的50万成年人（基线年龄30-79岁）。本研究纳入42,956名于2009至2017年间死亡的研究对象。 回家死亡定义为从医院出院后7天内在家中死亡。采用改良的泊松回归模型来估计死亡地点和回家死亡的现象随时间变化的趋势，并估计回家死亡与医保类型的关联，模型中调整社会人口学因素、根本死因和死亡年份等可能混杂因素。

**结果：**研究对象最常见的死亡地点是家中（71.5%），其次是医院（21.6%）。城乡居民基本医疗保险受益人回家死亡的比例约为城镇职工基本医疗保险受益人的6倍（66.0% vs 11.6%）。此外，无论是何种医疗保险的受益人，在生前最后一周回家死亡的比例均呈现增长趋势，城乡居民基本医疗保险受益人中年增长率为4.4%，城镇职工基本医疗保险受益人中年增长率为5.4%。与城镇职工基本医疗保险受益人相比，城乡居民基本医疗保险受益人更可能发生回家死亡，在调整社会经济学等潜在混在因素后，关联效应值现患比为1.19 （95%CI：1.12, 1.27）（P<0.001）。

**解读：**在中国人群中，最常见的死亡地点是家中。在生前最后一周有住院记录的研究对象中，很大一部分存在回家死亡现象，这种现象在城乡居民基本医疗保险受益人中尤为常见。不同医保类型的研究对象中从医院出院回家死亡的比例悬殊，表明不同医保的受益人在临终医疗资源利用方面可能存在不平等。
